# Shaping future leaders: developing an MPH leadership curriculum through problem-based learning

**DOI:** 10.3389/fpubh.2025.1612610

**Published:** 2025-07-18

**Authors:** Aimee McHale, Marie Lina Excellent, W. Oscar Fleming, Vaughn Mamlin Upshaw

**Affiliations:** Gillings School of Global Public Health, University of North Carolina at Chapel Hill, Chapel Hill, NC, United States

**Keywords:** leadership, curriculum and instruction, masters in public health, problem-based learning (PBL), workforce preparedness, collaborative problem solving, interdisciplinary

## Abstract

Leadership is essential to public health practice, yet few MPH programs offer structured, integrated approaches to cultivating leadership competencies. At the University of North Carolina’s Gillings School of Global Public Health, the Leadership in Practice (LIP) MPH concentration responds to this gap by embedding problem-based learning (PBL) throughout a multi-course curriculum designed to prepare students for real-world leadership challenges. Drawing on interdisciplinary faculty expertise and a constructivist pedagogical philosophy, the LIP curriculum emphasizes systems thinking, strategic decision-making, and values-based leadership development. This manuscript describes how PBL is applied across five required courses to help students synthesize technical knowledge with collaborative problem-solving and applied leadership skills. We also present alumni and employer feedback that illustrates the curriculum’s impact on workplace readiness and leadership capacity. This case study offers a model for integrating leadership development into graduate public health education and calls for broader adoption of applied, interdisciplinary pedagogies that prepare students to lead transformative change in diverse public health settings.

## Introduction: the urgent need for leadership in public health

Public health challenges today demand leaders who act decisively, think strategically, and engage diverse communities to drive systemic change. The COVID-19 pandemic, persistent and worsening health inequities, and the erosion of public trust in institutions have underscored the critical importance of capable public health leadership—not just at the top of agencies, but across all roles and sectors. Despite widespread acknowledgment of this need, leadership training remains inconsistently embedded in Master of Public Health (MPH) curricula domestically and globally (1, 2).

Calls for stronger public health leadership are not new (3, 4). The World Health Organization (5) and the Institute of Medicine (3) both emphasized the need to develop leadership capacity as essential to improving health outcomes and addressing health inequities. More recently, the American Public Health Association’s 2022 policy statement argued for a more community-centered, equity-focused model of leadership—one that departs from hierarchical norms and instead prioritizes collaboration, transparency, and systems thinking (6). Although several schools and programs of public health have created leadership courses, concentrations, or certificates, there is limited guidance on how best to teach leadership in a way that aligns with real-world practice. Often, leadership is taught as a discrete topic—an elective course, a short workshop, or a single module—rather than as an integrated component of the public health curriculum. This disconnection leaves students underprepared for the complex, interdisciplinary demands they will face in the field (2).

The Department of Public Health Leadership and Practice (PHLP) at the UNC Gillings School of Global Public Health sought to address this gap through the development of the Leadership in Practice (LIP) MPH concentration. This concentration is grounded in the belief that leadership is not a final stage in a career trajectory, but a practice—an orientation toward problem-solving, collaboration, and systems change that should be cultivated early and continuously. Central to the LIP curriculum is a pedagogical commitment to problem-based learning (PBL), a student-centered, experiential approach that mirrors the ambiguity, complexity, and collaborative nature of real-world public health practice (7).

This manuscript describes the rationale, design, and implementation of the Leadership in Practice MPH curriculum, with particular emphasis on how PBL supports the development of leadership competencies. We explore how interdisciplinary faculty collaborated to embed leadership development across a five-course sequence, examine the curriculum’s alignment with national competency frameworks, and present outcomes based on alumni and employer feedback. Our goal is to provide a model for other public health programs seeking to build applied, equity-oriented leadership capacity in their graduates.

## Leadership development in MPH programs: gaps and opportunities

While the need for effective public health leadership is well documented (4, 8, 9), the path to cultivating such leadership within graduate education remains uneven. Numerous national and international frameworks—including those from the Council on Education for Public Health (CEPH), the Council on Linkages Between Academia and Public Health Practice, and the de Beaumont Foundation—have outlined essential leadership competencies. These include systems thinking, strategic communication, stakeholder engagement, and policy advocacy (10–13).

Yet, many MPH programs struggle to integrate these competencies beyond the surface level (14, 15). For example, CEPH currently requires all MPH programs to address just two leadership competencies within their core curriculum (10). Although these are foundational, they are insufficient on their own to prepare students for the multidimensional leadership roles they will encounter in practice. Supplementary frameworks, such as the “Public Health Core Competency Domains” developed by the Council on Linkages (11) expand the scope but without a cohesive pedagogical strategy, these competencies risk becoming checkboxes rather than transformational learning goals.

Leadership education in public health often takes the form of isolated offerings—one-off workshops, elective courses, or extracurricular programs—rather than a sustained, scaffolded experience embedded across the curriculum. This fragmented approach does not reflect the reality of public health leadership, which requires continuous development across diverse contexts, teams, and systems (16). The urgency of this issue has grown in the wake of the COVID-19 pandemic, which accelerated workforce attrition and exposed leadership gaps across public health systems (17). An estimated one-third of local public health officials left their positions during or shortly after the pandemic. The profession now faces a generational shift, underscoring the need for training programs that not only replenish the workforce, but also reimagine how leadership is defined and taught (17, 18).

Emerging models of public health leadership emphasize relational and values-based skills—such as equity-centered decision-making, community engagement, and cultural humility—as essential complements to technical expertise. As articulated in a 2022 Tulane University blog and echoed in peer-reviewed literature, today’s leaders must be able to navigate ambiguity, build trust across sectors, and balance hard truths with optimism (15). Despite growing consensus about the value of leadership education, there is less agreement about *how* to teach it effectively. This creates an opportunity—and a responsibility—for schools of public health to innovate. At UNC-Gillings, the Leadership in Practice MPH concentration was developed to meet this need by offering a cohesive, interdisciplinary, and practice-based model for leadership education. Through the intentional use of problem-based learning (PBL), the LIP curriculum fosters critical, strategic, and adaptive skills that align closely with real-world public health leadership challenges.

## Pedagogical framework: problem-based learning for public health leadership

Effective public health leadership cannot be cultivated through passive learning or rote memorization. It must be developed through experience, reflection, and application—particularly in environments that simulate the ambiguity and complexity of real-world challenges. The Leadership in Practice (LIP) MPH concentration at UNC-Gillings is built on a constructivist pedagogical foundation, where students learn by engaging deeply with problems, collaborating across differences, and generating actionable solutions.

At the heart of this approach is problem-based learning (PBL), a student-centered methodology that begins with authentic, open-ended problems rather than predefined solutions. PBL is especially well-suited to leadership development because it cultivates the kinds of strategic and adaptive behaviors that leaders need in dynamic systems: critical thinking, team-based decision-making, systems analysis, and negotiation across sectors and stakeholder perspectives (19). Our faculty designed the LIP curriculum to align with constructivist principles drawn from Savery and Duffy (20) and Charlin et al. (21), emphasizing three pedagogical commitments:

1. *Learning through real-world engagement*: knowledge is best acquired through sustained interaction with complex, real-world challenges. Public health leadership requires grappling with uncertainty, conflict, and competing priorities. PBL immerses students in this reality by positioning them as co-creators of solutions in applied contexts.
2. *Dialogue and collaboration as learning mechanisms*: public health is inherently interdisciplinary. Leadership within it depends on communication, negotiation, and the ability to build shared mental models with diverse stakeholders. PBL fosters these skills by requiring learners to work collaboratively, give and receive feedback, and engage in reflective dialogue.
3. *Adaptability and meaning-making in uncertain contexts*: static, formulaic approaches cannot capture the complexity of public health systems. Instead, students must learn how to navigate ambiguity, reconcile competing values, and adapt to shifting conditions. PBL helps students develop the resilience and creativity needed to lead effectively in such contexts.

We also drew on insights from Ghani et al. (22), who identified three core themes of effective learning behavior in higher education settings where PBL is used:

- *Intrinsic*—students take initiative, manage timelines, and demonstrate persistence in the face of challenges.
- *Entrustment*—students assess their own performance, teach others, and seek feedback with openness.
- *Functional*—students prioritize issues, manage data, and collaborate effectively toward shared goals.

These themes map directly onto the leadership skills we seek to foster in the LIP concentration. By embedding them across five concentration courses and the culminating capstone project, we ensure that students do not simply learn about leadership—they practice it, reflect on it, and emerge with the strategic capacity to apply it in diverse public health settings. In the sections that follow, we describe how these pedagogical principles are implemented across the curriculum and illustrate how PBL is used to build leadership competencies at multiple levels: individual, organizational, and systemic.

## Curriculum design: the Leadership in Practice MPH concentration

The Leadership in Practice (LIP) concentration within the Master of Public Health (MPH) program at UNC Gillings School of Global Public Health was developed in response to a critical gap in graduate public health education: the lack of integrated, practice-based leadership training. Rather than treating leadership as a discrete topic or elective, the LIP concentration is built on the premise that leadership is a dynamic, applied practice—one that should be developed across the full span of a student’s training.

The LIP concentration is grounded in three interrelated commitments:

1. *Leadership as a way of working*: leadership is not defined by position or seniority. It is a way of approaching public health problems—with systems thinking, ethical clarity, and the ability to engage and mobilize others. This orientation is woven through the curriculum, beginning with values-based leadership and culminating in an integrative capstone experience.
2. *Problem-based learning as a core pedagogical strategy*: each required course in the concentration incorporates PBL principles, allowing students to engage deeply with real-world public health challenges. Through collaborative projects, case-based inquiry, and feedback-intensive assignments, students learn to think strategically, act collaboratively, and lead effectively across contexts.
3. *Interdisciplinary and practice-informed instruction*: the LIP curriculum is shaped by faculty with extensive experience in public, private, and nonprofit sectors. These “pracademic” instructors draw on their own leadership backgrounds in fields including public health, law, healthcare, policy, education, and engineering to design applied learning experiences that mirror professional demands.

### Curricular structure

All MPH students at UNC Gillings complete a 14-credit integrated core curriculum in their first year, which introduces foundational competencies required by the Council on Education for Public Health (CEPH) including epidemiology, biostatistics, health behavior, environmental health, health policy, nutrition and interprofessional practice. LIP students then take five required concentration courses across three semesters, followed by a culminating Integrated Learning Experience (ILE) capstone in their final semester.

The five required LIP courses include:

- Core Principles of Leadership in Public Health Practice
- Community Engagement and Leadership in Health
- Leading Continuous Quality Improvement (CQI) in Public Health Locally and Globally
- Leadership in Health Policy for Social Justice
- Systems and Design Thinking for Public Health Leaders

Each course is designed to reinforce specific competencies while progressively building students’ strategic and technical leadership capacities. The capstone course synthesizes learning across the curriculum, requiring students to apply leadership principles to a real public health challenge through a systems-change proposal. To ensure vertical integration and coherence across courses, faculty met regularly to align content, identify shared themes, and scaffold PBL assignments. This intentional collaboration supports not only curricular cohesion but also models the kind of cross-functional teamwork students are expected to practice.

## Application of problem-based learning across the LIP curriculum

Problem-based learning (PBL) serves as the pedagogical backbone of the Leadership in Practice (LIP) MPH concentration. Through carefully designed course sequences and assignments, students develop and apply leadership competencies in real-world public health contexts. Each of the five required concentration courses integrates core PBL themes—such as learner autonomy, collaborative inquiry, and real-world relevance—while aligning with specific leadership competencies. Rather than offering leadership training as abstract content, these courses challenge students to take on the role of practitioner-leaders. Working individually and in teams, students identify problems, assess context, design interventions, and reflect on outcomes. This scaffolded approach prepares them to lead across a variety of organizational, policy, and community settings.

Table 1 summarizes how each LIP course applies PBL principles and contributes to core leadership competencies.

**Table 1:** Leadership in practice MPH—course mapping to competencies and PBL principles.

Course	Leadership competency	PBL themes emphasized	key PBL features/assignments
Leadership in Public Health Practice	Integrate public health values and ethics in a philosophy of leadership; Propose structures of accountability for governance	Intrinsic, Entrustment	Systems change proposal using *Waters of Systems Change* (26); MOUs representing stakeholder roles
Community Engagement and Leadership in Health	Develop an inclusive strategy to engage with diverse stakeholders	Entrustment, Functional	Community asset mapping; collaborative stakeholder analysis
Leading CQI in Public Health	Apply appropriate methodologies to integrate research with practice-based evidence	Functional, Intrinsic	Real-world CQI case project with local health department; team-based process improvement plans
Leadership in Health Policy for Social Justice	Communicate effectively to promote a compelling public health agenda	Entrustment, Intrinsic	Policy brief development; peer feedback sessions on framing, values, and solutions
Systems and Design Thinking for Public Health Leaders	Design transformational systems to ensure effective public health practice	Functional, Intrinsic	Systems mapping and co-design strategies; team-based system redesign projects
Integrated Learning Experience	Synthesis of all LIP competencies through a real-world systems change proposal	All three themes	Independent project with team feedback; systems-level intervention planning

This structure reinforces a developmental arc across the curriculum. Early courses emphasize self-reflection and values-based leadership, while later courses shift toward systems thinking, co-design, and applied strategic planning. The capstone synthesizes these learning experiences by requiring students to lead a change process from diagnosis to proposal. By embedding PBL throughout the LIP curriculum, the program equips students not just with knowledge *about* leadership, but with the lived experience of *practicing* it—under uncertainty, in teams, and in service of equity and public impact.

### Collaborative faculty process: integration and interdisciplinary design

One of the distinguishing features of the Leadership in Practice (LIP) MPH concentration is the deeply collaborative process by which the curriculum was developed. Faculty from diverse disciplinary and professional backgrounds—public health, law, healthcare, engineering, policy, education, government and nonprofit management — came together with a shared goal: to design a curriculum that reflects the complexity of public health practice and prepares students to lead across systems. These faculty members, many of whom identify as *pracademics*—scholars with substantial applied experience—brought their field-based knowledge into the classroom and curricular planning process. They understood firsthand that public health leadership is rarely linear, never solitary, and often requires working across jurisdictions, organizations, and cultures. Their lived experience shaped both the content of the courses and the structure of the learning environment.

To ensure coherence and integration across the concentration, faculty engaged in sustained, iterative curriculum development. This involved:

- Aligning course objectives and assignments across semesters to support cumulative learning;
- Mapping competencies to assessments and PBL principles to ensure strategic skill development;
- Engaging in cross-course conversations about content overlap and how to reinforce key themes (e.g., equity, systems thinking, collaboration) without duplication; and
- Modeling collaboration by sharing teaching approaches, learning tools, and feedback across the team.

This design process mirrors the leadership behaviors the program seeks to cultivate in students: shared ownership, systems awareness, cross-boundary collaboration, and a commitment to continuous learning and improvement with community partners (23).

One innovative example of this collaborative philosophy in action is the incorporation of Collaborative Online International Learning (COIL) in the *Continuous Quality Improvement* course. Students from UNC and the Prasanna School of Public Health at Manipal Academy of Higher Education (India) collaborated asynchronously to co-develop quality improvement strategies for increasing mental health access in their respective countries. Despite the time zone difference and fully virtual format, students engaged in rich cross-cultural dialogue and co-created solutions that reflected diverse systems, priorities, and values. This experience reinforced the importance of adaptability, global awareness, and inclusive engagement—all critical to leadership in today’s interconnected public health landscape. A current Gillings student described the inter-institutional experience this way:

*“Working across borders helped me appreciate the shared humanity behind public health work, even when solutions must be culturally and contextually different. We became pros at asynchronous teamwork between coursework, flu season, and the infamous 9.5-h time difference. And yet, it worked—we created something thoughtful and informed using tools like Root Cause Analysis and PDSA cycles. Along the way, we shared life stories, laughed at tech issues, and built connections far beyond a Google Doc.” (UNC-Gillings student – Spring 2025).*

In essence, the curriculum development process modeled the very leadership practices it aims to teach. It demonstrated that building a transformative learning experience requires not only strong pedagogical design, but also intentional collaboration, a willingness to learn from one another, and an unwavering focus on real-world impact.

### Evidence of impact: alumni outcomes and leadership readiness

The Leadership in Practice (LIP) concentration was designed not only to teach leadership theories but to build students’ practical leadership capacity in real-world public health settings. To assess the program’s effectiveness, we examined multiple sources of outcome data, including alumni surveys, qualitative feedback, and employer perspectives. Together, these data points provide compelling evidence that the LIP curriculum prepares graduates for the complex leadership demands of public health practice.

#### Leadership training makes a measurable difference

Findings from the 2023 Gillings alumni survey revealed a significant gap in leadership preparation among MPH graduates across concentrations. Many respondents reported limited exposure to structured leadership development and expressed a desire for more applied training, particularly in decision-making, communication, and strategic thinking. In contrast, alumni from the Leadership in Practice concentration—captured in a 2025 alumni outcomes study (24)—described a markedly different experience. They credited the program’s interdisciplinary coursework, faculty mentorship, and problem-based assignments with preparing them to lead teams, navigate uncertainty, and drive organizational and community change. These alumni reported smoother transitions into leadership roles and higher confidence in their abilities to manage projects, influence policy, and collaborate across sectors.

#### Comparative analysis: alumni perspectives

Table 2 summarizes key differences between schoolwide survey respondents, most of whom lacked formal leadership training, and LIP concentration alumni.

**Table 2:** Comparative outcomes—leadership skills in the workplace.

Leadership skill area	General MPH alumni (survey)	LIP alumni (article/study)
Confidence in leadership roles	Felt underprepared; developed skills on the job through trial and error	Reported higher confidence and readiness to lead from day one
Decision-making	Lacked experience making high-stakes or ambiguous decisions	Gained tools for evidence-based, values-driven decision-making
Team management	Limited training in delegation, feedback, or collaboration	Learned team-building and communication through applied, group-based assignments
Strategic thinking	Unfamiliar with long-term planning or systems-level strategy	Practiced identifying leverage points and designing system-level interventions
Career advancement	Required additional on-the-job experience before assuming leadership roles	Progressed more rapidly into supervisory, directorial, or entrepreneurial positions

#### Beyond the classroom: alumni success stories

LIP alumni have advanced into prominent leadership roles across sectors. One such example is Dr. Diego Garza, a 2017 graduate, who now serves as Senior Vice President of Strategy and Innovation at Mindpath Health. His journey into leadership began with his practicum placement, later leading to full-time leadership in an organization that values innovation and cross-disciplinary collaboration. Alumni stories like Dr. Garza’s illustrate how applied, problem-centered learning builds a durable foundation for real-world leadership. A more recent alumnus in a leadership role reported that.

“*[p]ublic health leadership thrives on listening and collaboration, a principle I’ve witnessed firsthand. My education at UNC-Gillings and my daily practice have shown me that the most effective leaders are those who act according to their core values. In my role, I focus heavily on building relationships and facilitating collaboration, applying the leadership skills I’ve honed over time. My time at Gillings was invaluable for practicing public health skills. Public health is inherently a team effort, and reflecting on experiences like the capstone project, I recognize the importance of having a solid foundation in both technical and specific skills.”* (LIP alumnus, 2022)

#### Workforce readiness: skills beyond leadership

In addition to leadership capacity, alumni feedback also identified improvements in related skill areas often lacking in traditional MPH programs (25). These include communication, decision-making, and conflict resolution—skills central to leadership and essential for high-functioning public health teams (Table 3).

**Table 3:** Other workplace-relevant skills—reported alumni gaps vs. LIP gains.

Skill category	General MPH alumni (survey)	LIP alumni (article/study)
Communication skills	Needed more training in writing, presenting, and stakeholder dialogue	Gained practical experience crafting policy briefs, memos, and presentations
Problem-solving	Limited real-world case work; struggled to adapt skills	Used PBL to explore real health systems challenges and propose solutions
Conflict resolution	Insufficient training; reported discomfort in workplace negotiations	Learned collaborative problem-solving and feedback exchange in team settings

#### Global recognition and external validation

The program’s impact has also been recognized internationally. In March 2025, a UNC-Chapel Hill delegation presented the LIP alumni outcomes study at the National University of Singapore’s Saw Swee Hock School of Public Health. Senior faculty noted the depth and scope of UNC’s alumni tracking efforts (*n* = 49) and expressed interest in adopting similar approaches to measure workforce impact in their own programs. At the 2024 American Public Health Association (APHA) Annual Meeting, this study was presented as part of a panel featuring best practices in graduate and continuing education, further highlighting the relevance of and need for expanded leadership training in public health. Taken together, these outcomes demonstrate that the LIP concentration’s integrated, practice-based approach to leadership education not only prepares students to meet public health challenges but also positions them for early success and advancement in their careers.

## Discussion: lessons learned and implications for practice

The experience of developing and implementing the Leadership in Practice (LIP) concentration has yielded several key insights into the effective integration of leadership development within an MPH curriculum. This discussion reflects the practical implications of our approach, the challenges encountered, and the opportunities for continued innovation in public health education.

### Practical implications for leadership pedagogy

Our integrated, problem-based learning (PBL) approach underscores a fundamental shift in how leadership is taught. Rather than relying on isolated workshops or traditional lectures, embedding leadership in every aspect of the curriculum enables students to:

- *Build real-world competence:* by working on authentic challenges, students gain hands-on experience in navigating ambiguity, making evidence-informed decisions, and managing diverse teams.
- *Foster interdisciplinary collaboration:* the curriculum emphasizes cross-sector collaboration, reflecting the complex reality of public health practice. Through team-based projects and global partnerships (such as the COIL initiative), students learn to appreciate multiple perspectives and leverage collective expertise.
- *Enhance reflective practice:* continuous feedback and peer assessment build technical competence *and* cultivate the reflective habits necessary for lifelong leadership development.

### Challenges and limitations

Despite its many strengths, integrating a PBL approach into a broad MPH curriculum has presented several challenges:

- *Ensuring coherence across courses:* aligning course content while maintaining each course’s unique focus required sustained interfaculty dialogue and regular curriculum review sessions. Overcoming initial disparities in teaching philosophies was essential to create a seamless learner experience.
- *Time and resource intensiveness:* the extensive planning, coordination, and faculty training needed to implement PBL can be resource-intensive. Faculty members had to adapt to new roles as facilitators and mentors rather than traditional lecturers, a transition that demanded both time and institutional support.
- *Balancing flexibility with structure:* while PBL encourages learner autonomy, some students initially struggled with open-ended projects, and evidenced hesitation in dealing with ambiguity or the potential for failure. Balancing the structure of assignments with the necessary flexibility has been a continual learning curve, prompting ongoing refinement of rubrics and project guidelines.

### Future directions and recommendations

The insights gained from the LIP concentration suggest several avenues for further development:

- *Expanding collaborative networks:* strengthening partnerships both domestically and internationally can enrich the learning experience. Future iterations might include additional cross-cultural projects or real-time, virtual collaborative efforts that extend beyond the current COIL model.
- *Enhanced outcome measurement:* while initial alumni feedback and employer assessments are promising, a more systematic, longitudinal evaluation framework could better capture the long-term impact of PBL on leadership careers.
- *Normalizing ambiguity and struggle:* recognizing that students vary in their ability to navigate the kind of open-ended, complex problems featured in PBL, faculty and staff must be proactively engaged in clarifying and managing students’ expectations. Within the classroom, LIP faculty acknowledge student apprehension relative to the prospect of failure or “getting the wrong answer” while supporting students to learn through it. Within the department, intentional community building and mentoring foster a supportive, collaborative culture that normalizes struggle and supports risk-taking. These strategies and practices model leadership approaches that students can adopt and tailor with collaborators as they tackle similar complex challenges.
- *Continual faculty development:* sustaining the innovative teaching methods that define the LIP curriculum requires ongoing professional development, mentorship, and the establishment of communities of practice among faculty. Empowering instructors with regular training and shared best practices is critical for sustaining quality.
- *Adapting to evolving public health needs:* public health challenges evolve rapidly. As such, the curriculum must remain flexible enough to integrate emerging concepts, technologies, and approaches related to leadership and systems change. Periodic curriculum reviews and stakeholder input are vital to maintain relevance and responsiveness.

### A call for transformative leadership education

Ultimately, the LIP concentration demonstrates that leadership is not an endpoint, but a continuous process—an ever-evolving way of thinking and acting that must be cultivated through practical, applied experiences. By embedding leadership development into every facet of the MPH curriculum, UNC Gillings not only equips students with the tools they need today but also lays the foundation for the innovative, adaptive, and equity-driven leadership that public health increasingly requires. This model represents a call to action for public health programs worldwide to rethink traditional pedagogies and adopt integrated, experiential approaches that prepare graduates to lead transformative change.

## Conclusion: reimagining leadership education for public health impact

The challenges facing public health today—from persistent inequities to rapidly evolving global threats—require a new kind of leadership. Technical expertise alone is not enough. Public health professionals must also be prepared to lead strategically, ethically, and collaboratively across systems, sectors, and communities. This necessitates a fundamental rethinking of how leadership is taught and cultivated within schools and programs of public health.

The Leadership in Practice (LIP) concentration at UNC Gillings offers a model for meeting this challenge. Grounded in problem-based learning and shaped by faculty with deep practice-based experience, the LIP curriculum prepares students to navigate uncertainty, engage stakeholders, design solutions, and drive systems change. Leadership is not positioned as a final stage of development, but as a way of working that is integrated throughout a student’s training—beginning with values and self-awareness and culminating in real-world, systems-level impact.

By embedding leadership development across courses and competencies, and by emphasizing reflection, collaboration, and applied learning, the LIP concentration equips graduates with both the mindset and skillset needed to lead public health into the future. The evidence from alumni outcomes, employer feedback, and global recognition suggests this approach is not only innovative but effective.

As schools of public health reevaluate their educational models in response to workforce and societal demands, we offer this case study as both a practical example and a call to action. Leadership education should not be an add-on—it should be core. It should not wait until graduation—it should begin on day one. And it should not be theoretical—it should be lived, practiced, and continuously refined through real-world engagement.

The future of public health depends on the leaders we prepare today. Our goal is to help shape that future by offering a curriculum—and a pedagogical approach—that centers leadership as a public health imperative.

## Data Availability

The data analyzed in this study is subject to the following licenses/restrictions: internal alumni data collected by Gillings School of Global Public Health. Requests to access these datasets should be directed to Deytia Lima Rojas, PhD Assistant Dean for Strategic Analysis and Business Intelligence (SABI) UNC Gillings School of Global Public Health, Chapel Hill, NC, email: dlima@email.unc.edu.

## References

[1] Czabanowska K Kuhlmann E. Public health competences through the lens of the COVID-19 pandemic: what matters for health workforce preparedness for global health emergencies. Int J Health Plann Manag. (2021) 36:14–9. doi: 10.1002/hpm.3131, PMID: 33598987 PMC8013956

[2] Rodríguez-Feria P Czabanowska K Babich S Rodríguez-Sánchez D Carreño Hernández FL Hernández Flórez LJ. Divergence and convergence of the public health leadership competency framework against others in undergraduate medical education: a scoping review. Public Health Rev. (2023) 44:1605806. doi: 10.3389/phrs.2023.1605806, PMID: 37426906 PMC10323138

[3] Division of Health Care Services, & Committee for the Study of the Future of Public Health. The future of public health. London: National Academies Press (1988).

[4] Lachance JA Oxendine JS. Redefining leadership education in graduate public health programs: prioritization, focus, and guiding principles. Am J Public Health. (2015) 105:S60–4. doi: 10.2105/AJPH.2014.302463, PMID: 25706021 PMC4339997

[5] Czabanowska K. Leadership in public health: reducing inequalities and improving health. Eur Secur. (2014) 20:28–31.

[6] APHA. Reimagining public health leadership for health equity: Moving toward a collective and community-centered applied practice. Washington, DC: American Public Health Association (2022).

[7] Yew EH Goh K. Problem-based learning: an overview of its process and impact on learning, health professions. Education. (2016) 2:75–9. doi: 10.1016/j.hpe.2016.01.004, PMID: 40550732

[8] Rosenstock L Helsing K Rimer BK. Public health education in the United States: then and now. Public Health Rev. (2011) 33:39–65. doi: 10.1007/BF03391620, PMID: 32226193 PMC7099377

[9] Uno H Zakariasen K. Public health leadership education in North America. J. Healthcare Leadership. (2010) 22:11–5. doi: 10.2147/JHL.S9727

[10] CEPH. MPH foundational competencies. Washington, D.C.: Council on Education for Public Health (CEPH) (2021).

[11] Council on Linkages. Core Competencies for Public Health Professionals. Washington, D.C.: Council on Linkagees Between Academia and Public Health Practice (2021).

[12] deBeaumont Foundation and Association of State and Territorial Health Officials. Public Health Workforce Interests and Needs Survey, 2021 Dashboard. Bethesda, MD: deBeaumont Foundation and Association of State and Territorial Health Officials (2023).

[13] deBeaumont Foundation. Adapting and Aligning Public Health Strategic Skills. Bethesda, MD: deBeaumont Foundation and Association of State and Territorial Health Officials (2021).

[14] Begg MD Galea S Bayer R Walker JR Fried LP. MPH education for the 21st century: Design of Columbia University’s new public health curriculum. Am J Public Health. (2014) 104:30–6. doi: 10.2105/AJPH.2013.301518, PMID: 24228682 PMC3910049

[15] Tulane University Weatherhead School of Public Health and Tropical Medicine. Effective leadership in public health: Essential skills. New Orleans: Tulane University (2022).

[16] Helm-Murtagh S Erwin P. Building a new generation of public health leaders forged in a public health crisis. Am J Public Health. (2024) 114:626–32. doi: 10.2105/AJPH.2024.307633, PMID: 38603662 PMC11079828

[17] Brownson R Burke T Colditz G Samet J. Reimagining public health in the aftermath of a pandemic. Am J Public Health. (2020) 110:1605–10. doi: 10.2105/AJPH.2020.305861, PMID: 32816552 PMC7542265

[18] Meredith GR Welter CR Risley K Seweryn SM Altfeld S Jarpe-Ratner EA. Master of public health education in the United States today: building leaders of the future. Public Health Rep. (2023) 138:829–37. doi: 10.1177/00333549221121669, PMID: 36113136 PMC10467497

[19] Sherwood AL. Problem-based learning in management education: a framework for designing context. J Manag Educ. (2004) 28:536–57. doi: 10.1177/1052562904265773, PMID: 40550119

[20] Savery JR Duffy TM. Problem based learning: an instructional model and its constructivist framework. Educ Technol. (1995) 35:31–8.

[21] Charlin K Mann P Hansen B. The many faces of problem-based learning: a framework for understanding and comparison. Med Teach. (1998) 20:323–30. doi: 10.1080/01421599880742

[22] Ghani ASA Rahim AFA Yusoff MSB Hadie SNH. Effective learning behavior in problem-based learning: a scoping review. Med. Sci. Educ. (2021) 31:1199–211. doi: 10.1007/s40670-021-01292-0, PMID: 33903829 PMC8059994

[23] Archie-Booker E Osaji O Caldwell S Cooper P Garcia VN Waldrop R . Preparing learners for community-engaged public health practice. Am J Health Stud. (2020) 35:182. doi: 10.47779/ajhs.2020.201

[24] Excellent ML Yu M Hartranft A Fleming WO Ahsan KZ Schenck AP . Advancing pedagogy in leadership practice to enhance public health impact. J Public Health Manag Pract. (2025) 31:515–26. doi: 10.1097/PHH.0000000000002123, PMID: 40262185

[25] Burke EM Fox JA Turner B. Public health leadership: Enumeration and characterization of degree and certificate programs in schools and programs of public health. Washington, DC: ASPPH Center for Public Health Workforce Development (2025).

[26] Kania J Kramer M Senge P. The water of systems change. Boston, MA: FSG (2018).

